# P040: Epidemic of seasonal (2012-2013) influenza in a large teaching hospital

**DOI:** 10.1186/2047-2994-2-S1-P40

**Published:** 2013-06-20

**Authors:** A Iten, Y Thomas, C Landelle, V Camus, V Sauvan, L Kaiser, D Pittet

**Affiliations:** 1 Infection Control Program, University of Geneva Hospitals, Geneva, Switzerland; 2 Laboratory of Virology, University of Geneva Hospitals, Geneva, Switzerland

## Introduction

Seasonal influenza (SI) is usually a benign, self-limited disease of 2- to 7-day duration. However, it can present a serious threat to some patients, particularly those with underlying diseases, who may need hospitalization due to complications such as pneumonia. We describe an influenza epidemic in a 1900-bed Swiss university hospital from end December 2012 to mid-March 2013.

## Methods

Suspected cases of SI (respiratory symptoms, fever with chills, muscular pain, or prostration) were screened using nasopharyngeal samples and analyzed by real-time reverse transcriptase polymerase chain reaction. Patients’ clinical features and complications were evaluated**.** Cases were defined as nosocomial (NOSO) when symptoms occurred >72 h following admission.

## Results

Of 261 suspected cases, 171 (65.5%) samples were positive for influenza A and 90 (34.5%) for influenza B. Among these cases (median age, 76 y [range, 1.5-100.5]), 122 (46.7%) were male. A total of 117 (44.8%) were treated with oseltamivir and 38 (14.5%) were treated >72 h from disease onset. Clinical complications are still under investigation. Among these, 43 (16.5%) required intensive care and 9 (3.5%) died. A total of 94 cases were NOSO (36.1%). Hospital stay before NOSO SI diagnosis was 4 d for 2 cases, and >5 d for 92 cases. Recommended infection control procedures (droplet precautions with single room isolation whenever possible) were implemented for 230/261 patients (88.1%). Droplet precautions were applied for <3 days for 133/167 community-acquired SI (79.6%) cases and for 57/94 NOSO SI (60.6%). Among the NOSO SI, we observed 9 outbreaks involving 80 patients. Reliable information on vaccination status revealed that 53 patients were vaccinated. Healthcare workers’ vaccination status was low (~37%).

## Conclusion

Our prevention strategy against NOSO SI includes the weekly provision of SI epidemiological data to staff, and this information has been useful for the management of SI on the hospital wards. The epidemic is still active and data collection continues. The presented data will be updated to April 15, 2013.

## Disclosure of interest

None declared

